# A Study on Tencel/LMPET–TPU/Triclosan Laminated Membranes: Excellent Water Resistance and Antimicrobial Ability

**DOI:** 10.3390/membranes13080703

**Published:** 2023-07-28

**Authors:** Yue Zhang, Jia-Horng Lin, De-Hong Cheng, Xing Li, Hong-Yang Wang, Yan-Hua Lu, Ching-Wen Lou

**Affiliations:** 1 School of Chemical Engineering, Liaodong University, Dandong 118003, China; zhangyue19875@163.com (Y.Z.); chengdehongldxy1@163.com (D.-H.C.); 2 Liaoning Provincial Key Laboratory of Functional Textile Materials, Liaodong University, Dandong 118000, China; 3 Advanced Medical Care and Protection Technology Research Center, College of Textile and Clothing, Qingdao University, Qingdao 266071, China; jhlin@fcu.edu.tw; 4 School of Chinese Medicine, China Medical University, Taichung City 404333, Taiwan; 5 Advanced Medical Care and Protection Technology Research Center, Department of Fiber and Composite Materials, Feng Chia University, Taichung City 407102, Taiwan; 6 School of Textile Science & Engineering, Tiangong University, Tianjin 300387, China; simondongli@163.com; 7 Tianjing Fire Science and Technology Research Institute of MEM, Tianjin 300381, China; wanghongyang@tfri.com.cn; 8 Department of Bioinformatics and Medical Engineering, Asia University, Taichung City 413305, Taiwan; 9 Department of Medical Research, China Medical University Hospital, China Medical University, Taichung City 404333, Taiwan

**Keywords:** Tencel, low-melting-point polyester (LMPET), needle-punch, laminated nonwovens, thermoplastic polyurethane (TPU), protective properties

## Abstract

Medical product contamination has become a threatening issue against human health, which is the main reason why protective nonwoven fabrics have gained considerable attention. In the present, there is a soaring number of studies on establishing protection systems with nonwoven composites via needle punch. Meanwhile, the disadvantages of composites, such as poor mechanical performance and texture, impose restrictions. Hence, in this study, an eco-friendly method composed of needling, hot pressing, and lamination is applied to produce water-resistant, windproof, and antimicrobial Tencel/low-melting-point polyester-thermoplastic polyurethane/Triclosan (Tencel/LMPET–TPU/TCL) laminated membranes. Field-emission scanning electron microscope (SEM) images and FTIR show needle-punched Tencel/LMPET membranes successfully coated with TPU/TCL laminated membranes, thereby extensively improving nonwoven membranes in terms of water-resistant, windproof, and antimicrobial attributes. Parameters including needle punch depth, content of LMPET fibers, and concentration of TCL are changed during the production. Specifically, Tencel/LMPET–TPU/TCL–0.1 laminated nonwovens acquire good water resistance (100 kPa), outstanding windproof performance (<0.1 cm^3^/cm^2^/s), and good antimicrobial ability against *Escherichia coli* and *Staphylococcus aureus*. Made with a green production process that is pollution-free, the proposed products are windproof, water resistant, and antimicrobial, which ensures promising uses in the medical and protective textile fields.

## 1. Introduction

Nonwoven products comprise 60–70 percent of medical protection applications, and thus gain extensive attention. Nonetheless, owing to the low strength of the constituent fibers, nonwoven fabrics have lower mechanical performances that constrain their industrial uses. However, in a practical medical environment, medical practitioners are prone to contamination caused by bacteria, blood, and other pollutant fluids, and protective textiles are thus required to be antimicrobial and fluid-contamination-proof; to implement these, the majority of scholars coat or use other means to combine polymers and nonwoven matrices in order to expand the protection level of the nonwoven fabrics [1].

In the present day, PU showing various unique features, e.g., high elasticity, good tenacity, ultralightness, highly waterproof quality, and chemical-pollution-resistance properties has become one of the promising membrane materials [2,3,4,5]. Meanwhile, a growing number of antimicrobial PU membranes are becoming popular in the medical textile field. Most antimicrobial substrates can be used in the fabricating process, such as silver, quaternary ammonium groups, zinc oxide, chitosan, and triclosan [6,7,8]. Among antimicrobial agents, triclosan (TCL) exhibits the most potential due to its wide-spectrum antimicrobial activities [9]. For example, Zhao et al. [10] prepared a composite fibrous membrane containing antibacterial polyurethane (PU) by using an electrospining method; Dong et al. [11] created bilayered fibrous membranes consisting of an antibacterial agent and polymers; the electrospun dual-layer mats could continuously and spontaneously guide the directional water transport. Moustafa et al. [12] studied the feasibility of preparing antibacterial polyurethane composites for green packaging applications. In our previous study, TCL/TPU was employed to improve water protection and antibacterial activity [13]. The above studies are very significant because they are helpful in improving products’ antimicrobial abilities, but most scholars explore only woven/TCL antimicrobial and mechanical properties. In fact, composites that have only antimicrobial ability do not satisfy the practical applications of the medical environment. The potential of Tencel/LMPET–TPU/TCL composite membranes for producing protective wear with excellent water protection and windproof and antimicrobial performance has not been explored. In fact, coupling the multifunctional performances with composite membranes is a challenge because the physical properties of polymers and nonwovens are very different.

Based on the current studies, we propose a feasible method to generate laminated nonwovens with multiple functions; herein, the LMPET content as well as the triclosan content are changed in order to strengthen the protection performance. First, LMPET fibers with a sheath–core structure are used as reinforcement that is combined with Tencel fibers via hot pressing, forming Tencel/LMPET nonwoven fabrics. On this basis, they are combined and laminated with TPU/TCL compound membranes. Next, the morphology, FTIR, the water resistance, the windproof property, the texture, and the mechanical properties are compared systematically. The proposed laminated nonwovens have significant qualities, such as the use of industrial-grade materials, multiple protections of products, lower consumption of material, and feasibility of mass production, providing a new direction for developing medical and outdoor protective materials.

## 2. Experimental Section

### 2.1. Materials

Tencel^®^ fibers (known as Lyocel fibers, Haosen Fiber Technology Co., Ltd., Taichung City, Taiwan, China) have a fineness of 1.7 dtex and a length of 51 mm. Low-melting-point polyester fibers (LMPET fibers, Huvis Chemical Fiber Corporation, Seoul, Republic of Korea) have a fineness of 4 denier, a length of 64 mm, and a melting point of 110 °C. Thermoplastic polyurethanes (TPU, HV-7280EB, Headway Polyurethane Co., Ltd., Taichung City, Taiwan, China) have a melt index (MI) of 32 g/10 min (190 °C/8.7 kg). Triclosan (USP-K001.1) is provided by Taiwan Pharma-UP Enterprise Co., Ltd., Taichung City, Taiwan, China). PU resin is purchased from Twanfong Chemical Reagent Co., Ltd., Taichung City, Taiwan, China.

### 2.2. Preparation of Tencel/LMPET-TPU/Triclosan Laminated Membranes

**Figure 1:**
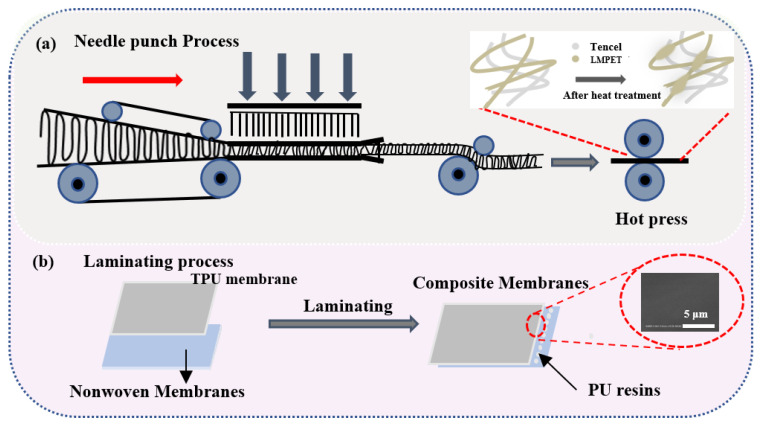
The manufacturing process of functional laminated nonwovens: (**a**) Tencel/LMPET nonwoven fabrics and (**b**) Tencel/LMPET–TPU/TCL laminated composite membranes.

To begin with, Tencel and LMPET fibers are blended at 100/0, 90/10, 80/20, 70/30, 60/40, and 50/50, and then the mixtures undergo opening, blending, laminating, and needle punching successively, producing Tencel/LMPET nonwoven fabrics with an areal weight of 130 ± 10 g/m^2^, a needle punch density of 200 needle/min, and a needle punch depth of 14 mm. Next, nonwoven fabrics are hot pressed at 160 °C at a rate being 7 rpm/min as seen in Figure 1a.

Next, the melt blending-coating method is used to produce TPU/triclosan compound membranes containing different triclosan contents. The temperature for the single-screw melt-blending process is 170 °C. Furthermore, Tencel/LMPET nonwoven fabrics and TPU/TCL membranes are bonded at 90 °C using PU resin. The resulting products are named Tencel/LMPET–TPU/TCL–x where x represents the triclosan contents, which are 0, 0.01, 0.05, 0.1, and 0.2, as shown in Figure 1.

### 2.3. Measurements and Characterizations

The morphology of membranes was characterized by a field-emission scanning electron microscope (FE-SEM, S-4800, HITACHI, Tokyo, Japan). The surface chemistry structure of the membranes was analyzed using a Fourier transform (FTIR) spectrometer (NICOLET iS10, Thermo Fisher Scientific, Waltham, MA, USA).

**Figure 2:**
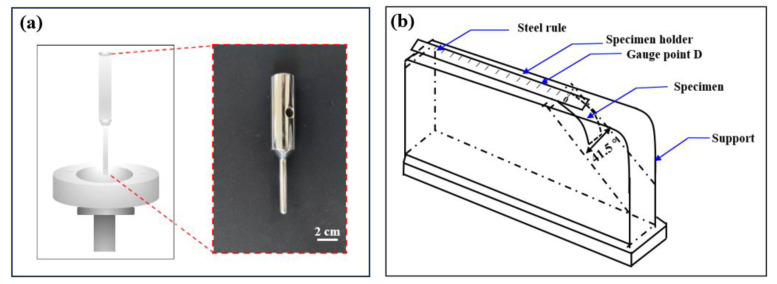
(**a**) Diagram of bursting strength test with the image of the bursting head. (**b**) Illustrative diagram of rigidity assembly.

A hydrostatic pressure test was used to evaluate the water resistance. Bursting strength (Figure 2a), tensile strength, flexibility (Figure 2b), and windproof properties were measured to evaluate the durability and comfort of the nonwoven membranes (Table 1). Membranes of tensile strength were taken along the machine direction (MD) and the cross machine direction (CD) separately for the test. A qualitative assessment antimicrobial testing was used to evaluate the membranes by observing the inhibition zones of two test strains: *Staphylococcus aureus* (ATCC25923) and *Escherichia coli* (ATCC25922). The protocol was performed according to the procedure described by Pinho et al. and Shiu et al. [14] Each sample was tested at least five times.

## 3. Results and Discussion

### 3.1. Mechanical, Breathable Properties, and Flexibility of Tencel/LMPET Nonwoven Fabrics

**Figure 3:**
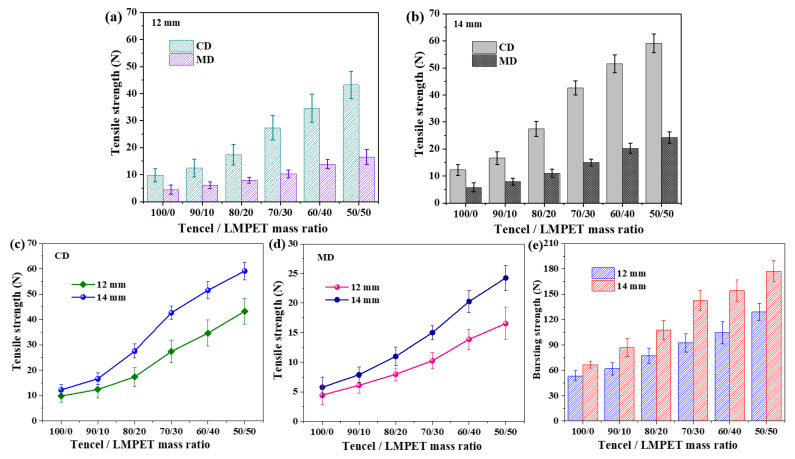
Tensile strength of Tencel/LMPET nonwoven fabrics as related to the needle punch depth of (**a**) 12 mm and (**b**) 14 mm as well as sample direction of (**c**) CD and (**d**) MD direction. (**e**) Bursting strength as related to ratio of LMPET fibers.

**Figure 4:**
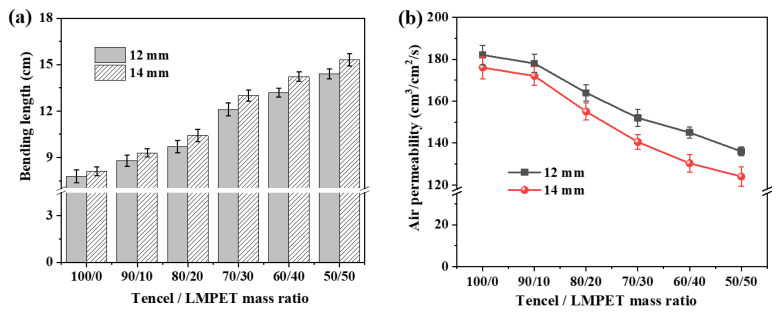
(**a**) Bending length and (**b**) air permeability of Tencel/LMPET nonwoven fabrics with various mass ratios of LMPET fibers.

Figure 3 and Figure 4 show the serial functions of Tencel/LMPET nonwoven fabrics, especially the mechanical properties that is the index of the service life of materials. According to our previous study, when LMPET fiber content exceeds 70%, the thermal bonding level among fibers reaches saturation. It is notable that 70% of LMPET fibers result in extreme rigidity and brittleness for nonwoven fabrics, and studies suggest that the appropriate an LMPET fiber content is 30–40 wt %. Therefore, LMPET fiber content is set between 0 wt % and 50 wt % in this study. Figure 4 shows the tensile strength of Tencel/LMPET nonwoven fabrics as related to the needle punch depth and the LMPET fiber content. Figure 3a,b show that either along the CD or the MD, a rise in the LMPET fiber content improves the tensile strength of nonwoven fabrics significantly because the sheath of LMPET fibers is melted to construct bonding points. As a result, the nonwoven density is increased, which enable Tencel/LMPET nonwoven fabrics to withstand an additional load.

As far as the test direction is concerned, regardless of the needle punch depth and LMPET fiber content, Tencel/LMPET nonwoven fabrics exhibit a greater tensile strength along the CD than the MD, which is ascribed to the needle punch machine and process. The discharge directions of the carding machine and needle punch machine are perpendicular. When fibers are arranged along the CD, they gain a greater contact area, and fibers can generate a greater friction along the direction of an external force. Therefore, Tencel/LMPET nonwoven fabrics exhibit a greater tensile strength along the CD [15]. In addition, Figure 3c,d demonstrate the effect of the needle punch depth on the mechanical properties of Tencel/LMPET nonwoven fabrics. The tensile strength of nonwoven fabrics is proportional to the needle punch depth because there is an increasing amount of vertical fiber bundles that enhances the entanglement and compactness among fibers. Afterwards, the employment of hot pressing contributes to more thermal bonding points and thereby achieving greater tensile properties. By contrast, pure Tencel nonwoven fabrics do not demonstrate the same trend in tensile strength as related to the needle punch depth. The tensile strength of pure Tencel nonwoven fabrics is not improved when the needle punch depth is increased. Although the needle punch depth only strengthens the entanglement level of fibers, thermal bonding points are absent in the pure Tencel nonwoven fabrics and result in limited reinforcement.

Figure 3e shows the bursting strength of Tencel/LMPET nonwoven fabrics as related to the needle punch depth and the LMPET fiber content. The bursting strength is improved when the LMPET fiber content increases. Moreover, a needle punch depth of 14 mm provides Tencel/LMPET nonwoven fabrics with greater bursting strength than a needle punch depth of 12 mm. Similarly, a greater needle punch depth generates a greater amount of vertical fiber bundles that help produce more bonding points during the hot pressing process, increasing the bursting strength considerably. Besides, functional nonwoven fabrics also demand good texture and a high comfort level. The incorporation of LMPET fibers effectively strengthens the mechanical properties of nonwoven fabrics, but the main challenge is the balance between the mechanical properties and the texture.

Figure 4a,b show the flexibility and windproof properties of Tencel/LMPET nonwoven fabrics as related to the needle punch depth and the LMPET fiber content. A rise in the LMPET fiber content has a positive influence on the bending length while adversely affecting the air permeability. The mechanism is that a greater number of thermal bonding points increases the fiber density. Comparing to the control group without hot pressing, the experimental groups are constrained with fiber slippage due to the presence of thermal bonding points. As a result, the more LMPET fibers, the more the thermal bonding points, and the less flexiblity the fibers gain. A needle punch depth of 14 mm, rather than 12 mm, generates a distinctively longer bending length for Tencel/LMPET nonwoven fabrics. Bending length (i.e., rigidity) and other mechancal properties share the same reliance on the thermal bonding points. Furthermore, as Figure 4b shows, Tencel/LMPET nonwoven fabrics containing more LMPET fibers show a higher windproof property, whereas nonwoven fabrics containing a smaller amount of LMPET fibers are more windproof. Based on the previous study, LMPET fibers could reduce the porosity of nonwoven fabrics because of the employment of hot pressing, causing significantly lower air permeability and a greater windproof property. Moreover, a needle punch depth of 14 mm provides the Tencel/LMPET nonwoven fabrics with a distinctively lower air permeability than a needle punch depth of 12 mm, which is ascribed to the same cause for lower mechanical properties. Therefore, with other parameters, the optimal Tencel/LMPET nonwoven fabrics attain synergistically improved machanical properties and flexibility when made of 40 wt % of LMPET fibers. This ratio of LMPET fibers is thus used for subsequent discussions.

### 3.2. Antimicrobial Activity Evaluation of the TPU/Triclosan (TCL) Membrane

Considering that biomaterials are prone to bacterial contamination, antimicrobial efficacy is indispensable for composite laminated nonwoven products when used clinically. Among tremendous antimicrobial agents, triclosan can effectively inhibit Gram-negative bacteria and Gram-positive bacteria when used with a low concentration. Therefore, triclosan is a highly efficient antimicrobial agent [16,17] and has been pervasively used in the industrial products for it meets the European Union and US requirements [18,19,20,21]. It is notable that the aforementioned standards demand that the dosage of triclosan needs to be lower than 0.3 wt %. As a result, current studies exploring antimicrobial efficacy only use 0.2 wt % triclosan.

**Figure 5:**
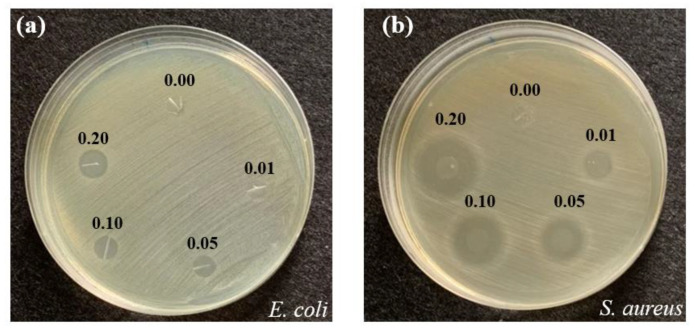
Antimicrobial activity of TPU membrane with different triclosan concentrationsagainst: (**a**) *E. coli* and (**b**) *S. aureus*.

Figure 5 shows that pure TPU membranes barely show an inhibition zone. Because of an insufficient amount of triclosan, TPU-TCL-0.01 and TPU-TCL-0.05 membranes are not effective in destroying harmful Gram-negative *E. coli* bacteria; the former does not exhibit an inhibition zone while the latter shows an insignificant level of antimicrobial performance. Conversely, TPU-TCL-0.1 and TPU-TCL-0.2 display stable and distinctive antimicrobial performance. As far as Gram-positive *S. aureus* bacteria are concerned, TPU-TCL-0.01 is capable of killing and reducing the bacteria, whereas pure TPU membranes fail to generate an inhibition zone. The membranes have increasingly strengthened antimicrobial efficacy as a result of a rise in triclosan.

TPU/TCL membranes possess good antimicrobial effect against *E. coli* and *S. aureus,* especially the latter. Interestingly, TPU/TCL membranes have much greater antimicrobial effect against *S. aureus* than against *E. coli*, as substantiated by a wider inhibition zone. In terms of bacterial type, *E. coli* is Gram-negative and *S. aureus* is Gram-positive. The difference in sensitivity between Gram-negative and Gram-positive bacteria is probably due to the different organization of the cellular structure. The cell membrane of Gram-negative bacteria is composed of lipopolysaccharides, lipids, and protein, which leads to a narrower inhibition zone [22,23,24,25]. To sum up, based on the specified manufacturing parameters, considering the stable and reproductive antimicrobial effect, TPU-TCL-0.1 is the optimal group and used for subsequent examinations.

The membranes are responsive differently to Gram-negative and Gram-positive bacteria. Composed of lipopolysaccharides, lipids, and protein, Gram-negative bacteria have a different cell structure from *S. aureus*, and thus causes a narrower inhibition zone [22,23,24]. To sum up, based on the specified manufacturing parameters, considering the stable and reproductive antimicrobial effect, TPU-TCL-0.1 is the optimal group and used for subsequent examinations.

### 3.3. Morphology and FTIR of the Lamination Composite Membranes

**Figure 6:**
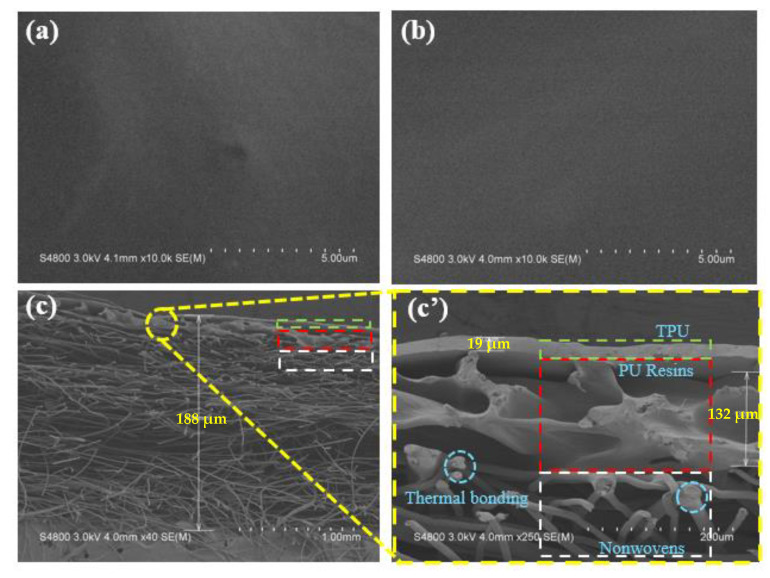
SEM image of (**a**) Tencel/LMPET–TPU/TCL–0 membranes, (**b**) Tencel/LMPET–TPU/TCL–0.1 composite membranes, (**c**,**c’**) Cross-section image of Tencel/LMPET–TPU/TCL–0.1 composite membranes.

In this study, Tencel/LMPET nonwoven fabrics are made in a specified ratio of 60/40, which means there are 40 wt % of LMPET fibers. According to the previous discussion, TPU/TCL membranes containing 0.1 wt % of triclosan (TPU/TCL-0.1) are optimal. In this section, there are three sample groups, involving the control group (pure composite nonwovens) and the experimental groups (nonwoven-membrane, containing 0 and 0.1 wt % of triclosan). The samples are named according to the constituent materials, which are Tencel/LMPET nonwovens, Tencel/LMPET–TPU/TCL–0, and Tencel/LMPET–TPU/TCL–0.1, respectively. The SEM images in Figure 6a–c show the morphology of laminated nonwovens as related to three triclosan contents. Figure 6 clearly shows the structure of pure TPU membranes. With the presence of triclosan, TPU membranes retain the structure without any remarkable difference, regardless of whether it is in Tencel/LMPET–TPU/TCL–0.1 or Tencel/LMPET–TPU/TCL–0. Furthermore, Figure 6c,c’ shows the cutting section of Tencel/LMPET–TPU/TCL–0.1 where the three constituent layers are laminated from top to bottom in order, including a Tencel/LMPET nonwoven fabric, PU resin, and a TPU/TCL-0.1 membrane. Simply put, the top and bottom layers are firmly attached with the PU interlayer. Moreover, the thickness is 132 μm for the PU resin and 19 μm for the TPU/TCL membranes.

**Figure 7:**
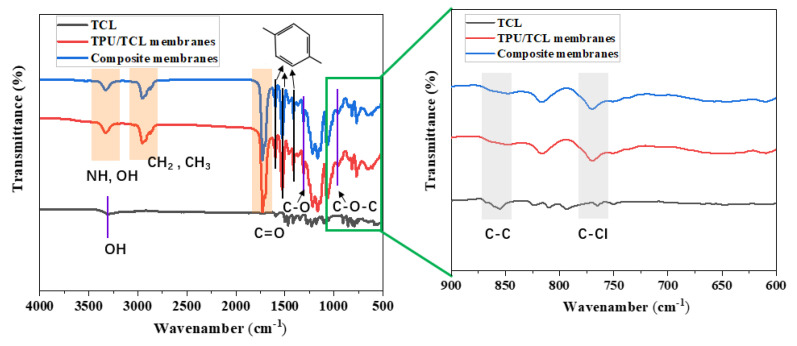
FTIR spectra of TCL, TPU/TCL and Tenecel/LMPET–TPU/TCL composite membranes.

Figure 7 shows the FTIR spectra of the TCL, TPU/TCL membranes and Tenecel/LMPET-TPU/TCL composite membranes. The bands at 2946 cm^−1^ and 2856 cm^−1^ demonstrate the presence of symmetric and asymmetric stretching of the –CH_2_ and –CH_3_ groups [26,27]. The characteristic carbonyl-stretching (C=O) absorption peak of PCL at 1728 cm^−1^ is presented for all the spectra of TPU/TCL and composite membranes [28]. Peaks at 1406, 1520, and 1619 cm^−1^ originate from the benzene ring C=C group of TPU [29]. The bands at 1311 and 997 cm^−1^ are attributed to C–O–C and C–O, respectively. The TPU/TCL and composite membranes exhibit typical –NH peak characteristic bands at 3334 cm^−1^ for the stretching vibration of the amide group [30]. There are specific absorption peaks at 3300 cm^−1^ related to –OH bonds in TCL [31]. As shown in spectra between 900–600 cm^−1^, the peaks at 762 cm^−1^ and 864 cm^−1^ indicate C–Cl bonds and C–C [9]. The FTIR spectra indicate that TPU/TCL membranes has been successfully deposited onto the nonwovens after lamination.

### 3.4. Water Resistance and Machanical Properties of the Lamination Composite Membranes

**Figure 8:**
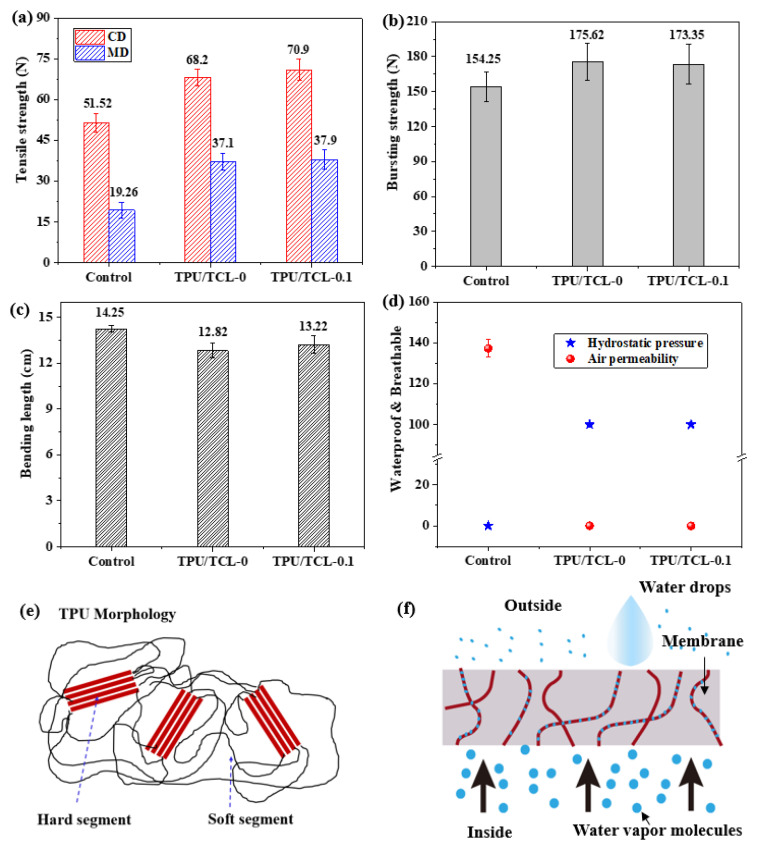
(**a**) Tensile strength, (**b**) busting strength, (**c**) bending length, and (**d**) waterproof & breathable performance of the control group, Tencel/LMPET–TPU/TCL–0, and Tencel/LMPET–TPU/TCL–0.1 laminated nonwovens. Schematic illustrations of (**e**) structure of TPU and (**f**) water resistance of TPU/TCL membrane.

Figure 8a–f demonstrate the mechanical properties of laminated nonwovens. Regardless of the triclosan content, the experimental groups have greater strengths than the control group (i.e., Tencel/LMPET nonwoven fabrics). This result can be attributed to the structure of laminated nonwovens as seen in Figure 6c. As for the control group, fibers are entangled and bonded by the melted sheath of LMPET fibers, indicated by a dotted blue circle in Figure 6c’. The damage mechanism of a tensile force is mainly based on the sliding fibers for pure nonwoven fabrics, which is totally different from that of laminated nonwovens. By contrast, laminated nonwovens consist of PU resin and TPU membranes via different combinations between heterogeneous polymers, which in turn causes different mechanical responses and deformation [32]. Moreover, PU resin creates tremendous solidifying bonds that immobilize some portions of the fibers, with flexible TPU membranes being bonded as a protective layer, providing laminated nonwovens with toughness and strength to withstand extra expansion. Concurrently, the presence of nonwoven fabrics serves as a supportive source, improving the mechanical properties effectively. In addition, Tencel/LMPET–TPU/TCL–0 and Tencel/LMPET–TPU/TCL–0.1 show comparable tensile strength, which is in conformity with the result of a previous study [33]. Figure 8b shows the bursting strength of the control group (i.e., Tencel/LMPET nonwoven fabrics) and experimental groups (i.e., Tencel/LMPET–TPU/TCL–0, and Tencel/LMPET–TPU/TCL–0.1 laminated nonwovens). It is apparent that the laminated nonwovens outperform pure nonwoven fabrics in terms of bursting strength, which is ascribed to the same damage mechanism discussed for tensile strength, and so the TPU protective layer enhances the bursting strength. As the incorporation of triclosan with laminated nonwovens does not affect the morphology of the products (Figure 6), triclosan is irrelevant with the bursting strength.

In addition to the mechanical properties, outdoor and medical products demand water resistance and flexibility, too. Figure 8c shows that the experimental groups still possess greater flexibility than the control group, which is also attributed to the resilient TPU membrane. Unlike the control group, which is without the waterproof attribute, the experimental groups exhibit water resistance as high as 100 kPa (Figure 8d), the mechanisms of which are shown in Figure 8e,f. The hydrophobic properties of solid surfaces are related to the surface chemical composition and microstructure, such as whether it is a rough surface or not. For the chemical composition, TPU is a hydrophobic elastic polymer with soft segments and hard segments, and its membranes do not have pores (as seen in Figure 6), which in turn provide laminated nonwovens with high water resistance and windproof functionality. Furthermore, the water resistance mechanism can be expained by the Laplace–Young equation, which was correlated to the membrane pores and water surface tension (γ). In comparison with the control nonwovens, the introduction of a TPU hydrophobic film changes the surface tension of the laminate composite membranes, thus improving the hydrophobic property [34,35,36,37]. The water resistance and antimicrobial property of the proposed laminated nonwovens (Tencel/LMPET–TPU/TCL–0.1) are compared with the results of a previous study [2,3,4,11,34,38,39,40,41,42,43] (as seen in Table 2), in which the hydrostatic pressure was 15–84 kPa for PU-contained laminated fabrics and 38–83 kPa for electrospinning nonwoven membranes. By contrast, Tencel/LMPET–TPU/TCL–0.1 laminated nonwovens show a greater hydrostatic pressure of 100 kPa because of LMPET fibers and compact PU pore-free membranes. The proposed laminated nonwovens also receive a generally favorable water resistance via the employment of needle punch, hot press, full cure of PU resin, and lamination.

## 4. Conclusions

A Tencel/LMPET–TPU/TCL composite membrane was successfully prepared via a simple needle punch and lamination method. The improved mechanical properties of the nonwoven membranes could be achieved with an increase in LMPET ratio. The test results indicate that a needle punch depth of 14 mm, LMPET fiber content of 40%, and TCL concentration of 0.1% contribute to an outstanding water resistance of 100 kPa, an excellent windproof property that is lower than 0.1 cm^3^/cm^2^/s, and good antimicrobial ability against *E. coli* and *S. aureus*. These experimental results are quite significant, considering the lower consumption of antimicrobial agent, easier processing, and multiple protections. The cooperative strategy of needle punch and lamination is suitable for large-scale production.

## Figures and Tables

**Table 1:** Test on the membrane specimens.

Test Name	Test Criterion	Instrument	Sample Size, cm
Hydrostatic pressure	AATCC 127	YG812, Nantong Hongda Experiment Instruments Co., Ltd., Nantong, China	circular with a diameter of 100
Bursting Strength	ASTM F2054-07	Instron 5565, Instron, Boston, MA, USA	15 × 15
Tensile strength	ASTM D5035-11	Instron 5565, Instron, Boston, MA, USA	180 × 2.54
Windproof performance	ISO 9273:1995	TEXTEST FX3300, Aidengwei Automation Instruments, Shanghai, China	25 × 25
Flexibility	GB/T18318.1-2009	DRK207BDRK, DRK Experiment Instruments Co., Ltd., Shandong, China	2.5 × 25
Antimicrobial properties	AATCC 90-2011	Solid agar Petri plate, Hengfeng Medical Instruments Co., Ltd., Huangshi, China	circular with a diameter of 1

**Table 2:** Comparison of the water resistance and antimicrobial performances between this study and previous studies.

Substrate	Modifacation Materils	Treatment Method	Hydrostatic Pressure, kPa	**Antimicrobial Activity**		Ref.
				***E. coli***	***S. aureus***	
Tencel/LMPET@PU	PU/TCL	Lamination	100	○	○	This work
Polyester@PU	PU	Lamination	78	×	×	[2]
Taslan@PU	PU	Lamination	84	×	×	[3]
Cotton@PU	PU	Lamination	52	×	×	[4]
PAN@PVDF/ZnO	PVDF/ZnO	Electrospun	—	○	×	[11]
Nylon@PU	PU	Lamination	32	×	×	[34]
Nylon@WPU	WPU	Coating	15	×	×	[38]
PVDF/PVB	PVB	Electrospinning	58	×	×	[39]
PAN/WFPU	WFPU	Electrospinning	83	×	×	[40]
PVDF/PU/PVDF	PVDF	Electrospinning	38	×	×	[41]
PET/VIS/cotton@AgCl	AgCl	Spraying	—	○	○	[42]
Tencel/cotton@propoils	propoils	Dip coating	—	○	○	[44]

Where ○ stands for the antimicrobial property and × refers to non antimicrobial property. PVDF: polyvinylidene fluoride; PVB: polyvinyl butyral; PU: polyurethane; WPU: waterborne polyurethane; PAN: polyacrylonitrile.

## Data Availability

Not applicable.
